# Evaluation of the LDN-0060609 PERK Inhibitor as a Selective Treatment for Primary Open-Angle Glaucoma: An In Vitro Study on Human Retinal Astrocytes

**DOI:** 10.3390/ijms25020728

**Published:** 2024-01-05

**Authors:** Wioletta Rozpędek-Kamińska, Grzegorz Galita, Kamil Saramowicz, Zuzanna Granek, Julia Barczuk, Natalia Siwecka, Dariusz Pytel, Ireneusz Majsterek

**Affiliations:** 1 Department of Clinical Chemistry and Biochemistry, Medical University of Lodz, 90-419 Lodz, Poland; wioletta.rozpedek@umed.lodz.pl (W.R.-K.); grzegorz.galita@umed.lodz.pl (G.G.); kamil.saramowicz@stud.umed.lodz.pl (K.S.); zuzanna.granek@stud.umed.lodz.pl (Z.G.); julia.barczuk@stud.umed.lodz.pl (J.B.); natalia.siwecka@stud.umed.lodz.pl (N.S.); 2 Department of Pathology and Laboratory Medicine, Medical University of South Carolina, Charleston, SC 29425, USA; pytel@musc.edu

**Keywords:** eIF2α, endoplasmic reticulum stress, glaucoma, glaucoma treatment, PERK, PERK inhibitor, unfolded protein response

## Abstract

The term glaucoma encompasses various neurodegenerative eye disorders, among which the most common is primary open-angle glaucoma (POAG). Recently, the essential role of human retinal astrocytes (HRA) in glaucoma progression has been placed in the spotlight. It has been found that placing the endoplasmic reticulum (ER) under stress and activating PERK leads to apoptosis of HRA cells, which inhibits their neuroprotective effect in the course of glaucoma. Therefore, the aim of the present study was to evaluate the effectiveness of the small-molecule PERK inhibitor LDN-0060609 in countering ER stress conditions induced in HRA cells in vitro. The activity of LDN-0060609 was studied in terms of protein and mRNA expression, cytotoxicity, genotoxicity, caspase-3 level and cell cycle progression. LDN-0060609 at 25 μM proved to be a potent inhibitor of the major PERK substrate, p-eIF2α (49% inhibition). The compound markedly decreased the expression of pro-apoptotic ER stress-related genes (*ATF4*, *DDIT3*, *BAX* and *Bcl-2*). Treatment with LDN-0060609 significantly increased cell viability, decreased genotoxicity and caspase-3 levels, and restored cell cycle distribution in HRA cells with activated ER stress conditions. These findings indicate that the small-molecule PERK inhibitor LDN-0060609 can potentially be developed into a novel anti-glaucoma agent.

## 1. Introduction

*Glaucoma* is an umbrella term for a heterogeneous group of chronic eye diseases characterized by increased intraocular pressure, neurodegeneration of the optic nerve, and rapid loss of retinal ganglion cells (RGC), leading to progressive vision loss and, ultimately, irreversible blindness [1]. The number of glaucoma patients is constantly growing and is estimated to reach 111.8 million worldwide in 2040 [2]. Clinically, glaucoma can be subdivided into open-angle glaucoma (OAG) and angle-closure glaucoma (ACG), based on the morphology of the anterior chamber angle. Both OAG and ACG can be classified as primary, i.e., occurring without an identifiable cause, or secondary, i.e., resulting from a known underlying factor, condition or trauma [1]. Of these, primary open-angle glaucoma (POAG) is the most common type, accounting for almost 80% of all glaucoma cases worldwide [2]. Although glaucoma is currently the most frequently researched cause of visual impairment [3], neither the molecular mechanisms leading to its development nor the factors contributing to its progression have been fully elucidated.

Recent data provide compelling evidence linking endoplasmic reticulum (ER) stress and the unfolded protein response (UPR) with the pathogenesis of glaucoma [4,5]. The ER is a high-capacity molecule-modifying compartment that plays a crucial role in maintaining cellular homeostasis [6]. Any disruption in its homeostasis, induced inter alia by the accumulation of misfolded proteins in the ER lumen or elevated intraocular pressure (IOP), a characteristic feature of glaucoma, increases ER stress [7]. In fact, the presence of the best-known POAG-related mutation, *viz. MYOC* in the myocilin gene, leads to abnormal folding and aggregation of mutant myocilin [8]. Elevated ER stress results in the activation of three major signaling cascades initiated by ER trans-membrane protein sensors: protein kinase R (PKR)-like endoplasmic reticulum kinase (PERK), inositol-requiring enzyme 1 alpha (IRE1α) and activating transcription factor 6 (ATF6). The UPR acts to restore cellular homeostasis; however, prolonged ER stress results in the activation of the pro-apoptotic branch of the UPR pathway, in which a key role is played by the PERK-dependent pathway [9]. PERK activation leads to the phosphorylation of the eukaryotic initiation factor 2α (eIF2α), which subsequently downregulates global protein translation; at the same time, it stimulates the translation of specific mRNAs and proteins, such as activating transcription factor 4 (ATF4). Under mild and intermediate ER stress, enhanced ATF4 translation is able to restore cellular homeostasis. However, prolonged ER stress leads to apoptotic cell death mediated by DNA damage-inducible transcript 3 (DDIT3), also known as CCAAT-enhancer-binding protein homologous protein (CHOP), and its downstream factors such as B-cell lymphoma-2 (Bcl-2) family members [9]. 

Recently, attention has been brought to the role of glial cells in the pathogenesis of numerous neurodegenerative diseases, including glaucoma. The most prevalent type of glial cell in the central nervous system (CNS) is the astrocyte, which constitutes the main cellular component of the optic nerve head (ONH), a key site of early pathological events observed in glaucoma [10]. Human retinal astrocytes (HRA) play numerous vital roles in the human retina, and are essential for maintaining RCG homeostasis [11]. Astrocytes provide neurons with essential nutrients, such as lactate and amino acids, and are able to stimulate the regeneration of the axons of the RGC via astrocyte-derived ciliary neurotrophic factors [12,13]. The RGC axons require structural and physiological support from HRAs, especially in the ONH, where RGC axons are unmyelinated, and due to the passive conduction of action potentials, they display high metabolic demands [14]. Furthermore, HRAs are also involved in the development of the retinal vascular system and the formation of the blood–retinal barrier [11]. Together with Müller cells, the largest glial cell in the retina, astrocytes ensure the integrity of the blood–retinal barrier by balancing the tight junctions between endothelial cells and preserving the immune privilege of the eye [15]. It has been suggested that in the early stages of glaucoma, HRAs may promote neuronal survival and prevent progressive neurodegeneration. However, upon prolonged IOP and ER stress, HRA cells may transition to a reactive state and produce numerous neurotoxic factors, resulting in axonal damage and exacerbating glaucoma [16,17,18]. Moreover, there is evidence linking astrocyte remodeling and neuropathy progression in the optic nerve, and HRA overactivity linked to glaucoma has been demonstrated both in animal models as well as in humans [10].

Multiple studies have addressed the importance of PERK/elF2α/ATF4/DDIT3 axis inhibition as a promising target for restoring the proper function of TM cells and preventing apoptosis of RGCs [19,20,21,22]. However, there is a dearth of knowledge on the outcome of ER stress inhibition in retinal astrocytes. Hence, considering the potential contribution of HRA to glaucoma pathogenesis, the aim of the present study was to determine the effectiveness of LDN-0060609 in preventing the negative outcomes of ER stress in HRA cells in vitro, and to further characterize its potential application in POAG treatment.

## 2. Results

### 2.1. The Effect of the LDN-0060609 PERK Inhibitor on HRA Cells with Activated ER Stress Conditions

eIF2α is the major substrate of PERK, and is directly phosphorylated by PERK upon activation of ER stress conditions [23]. The activity of LDN-0060609 was determined by measuring the level of p-eIF2α (phosphorylated form of eIF2α) by Western blot. For this purpose, HRA cells were preincubated with the LDN-0060609 PERK inhibitor at a range of concentrations (0.75–50 μM) for one hour; they were then treated with an ER stress inducer, Th, at 500 nM, and incubated for two hours. The positive control consisted of cells incubated with 500 nM Th for two hours only, and the negative control of those cultured in complete AM cell culture medium only. 

**Figure 1:**
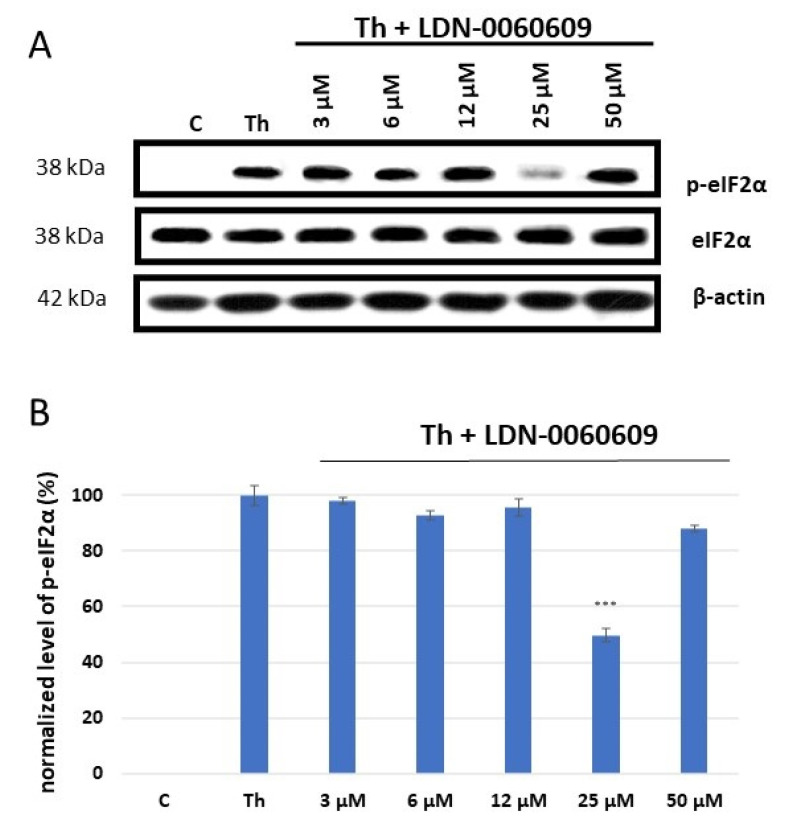
Western blot analysis (**A**) and quantification measured by means of optical densitometry (**B**) of eIF2α phosphorylation in HRA cells treated with the LDN-0060609 PERK inhibitor and Th (thapsigargin) as an ER stress inducer. All experiments were performed in triplicate, values are expressed as mean ± SEM, *n* = 3. *** *p* < 0.001 versus the positive control (Th). C—negative control (untreated HRA cells); Th—thapsigargin-treated HRA cells (ER-stressed HRA).

Th effectively induced ER stress in HRA cells: the level of p-eIF2α was significantly elevated in HRA cells incubated with 500 nM Th in comparison with control HRA cells, i.e., those cultured in complete medium only. Furthermore, a significant decrease in ER stress-dependent eIF2α phosphorylation was found in HRA cells under ER stress conditions (ER-stressed HRA cells) caused by treatment with LDN-0060609 PERK inhibitor. The obtained results indicate that LDN-0060609 had the highest activity in ER-stressed HRA cells at a concentration of 25 μM, with LDN-0060609 significantly inhibiting eIF2α phosphorylation by 49% (Figure 1).

### 2.2. Evaluation of mRNA Expression via ER Stress-Related Genes in HRA Cells Treated with LDN-0060609 

**Figure 2:**
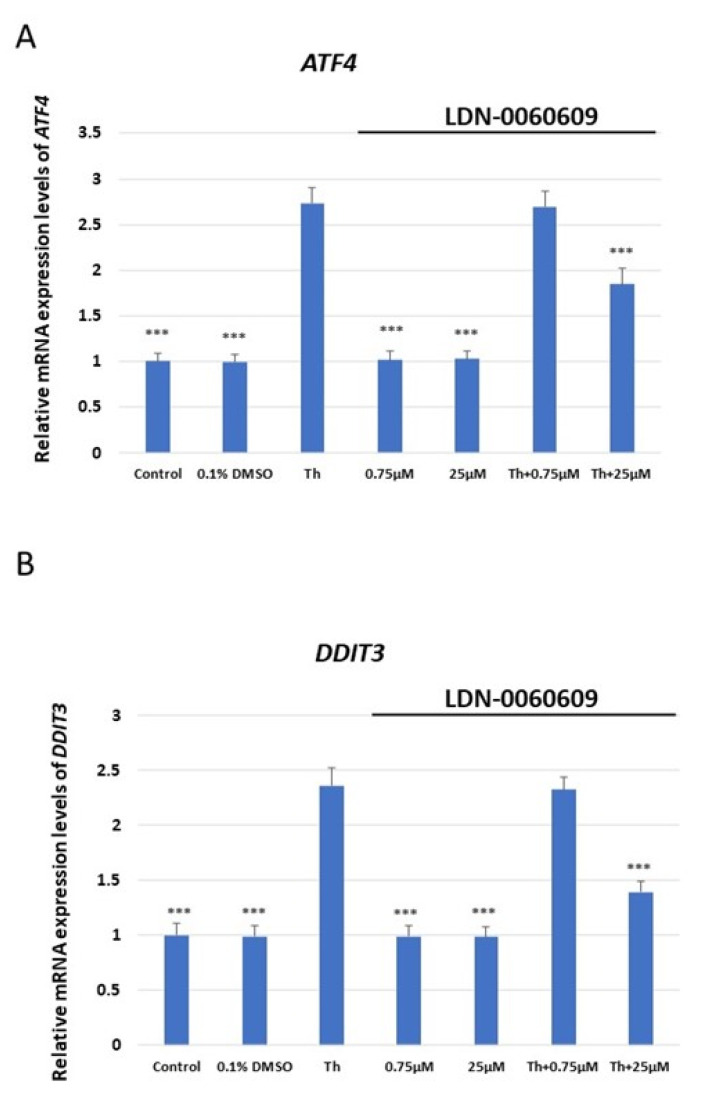

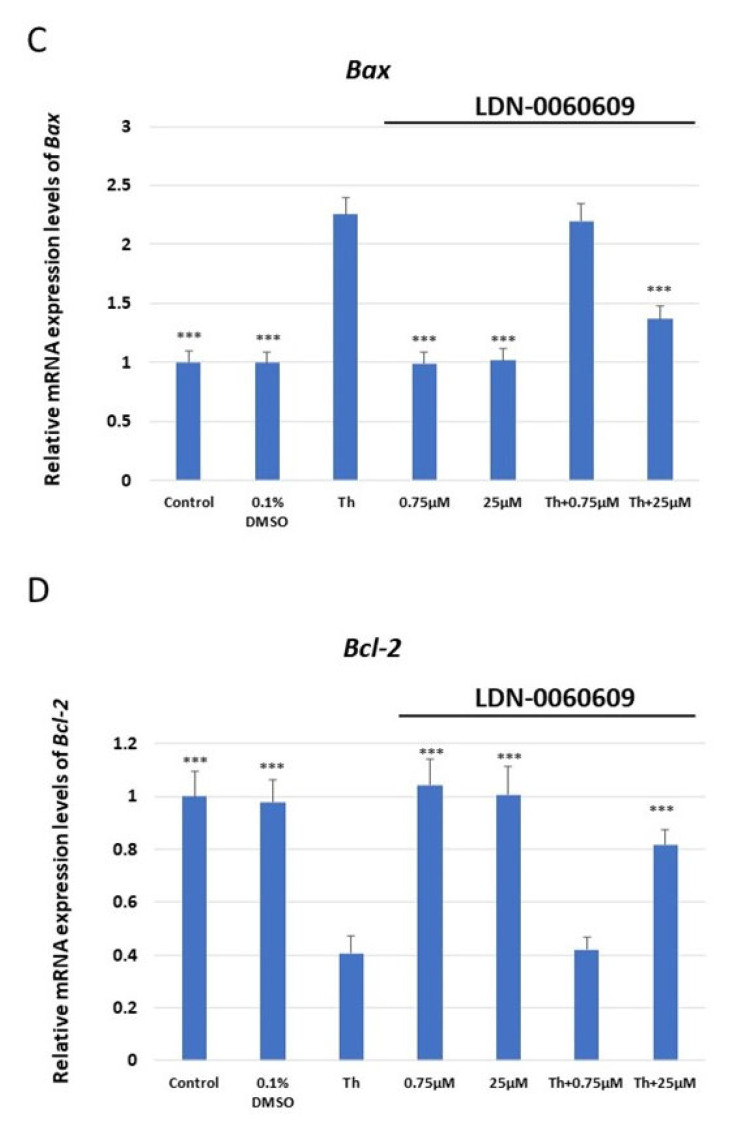
Evaluation of the mRNA expression of the ER stress-related apoptotic genes: (**A**) *ATF4*, (**B**) *DDIT3*, (**C**) *BAX*, (**D**) *Bcl-2* in HRA cells treated with the small-molecule PERK inhibitor LDN-0060609 alone, Th alone, or with both Th and LDN-0060609. The analysis was performed using a TaqMan gene expression assay. All experiments were performed in triplicate, and values are expressed as mean ± SEM, *n* = 3. *** *p* < 0.001 versus Th. Control—untreated HRA cells; 0.1% DMSO—HRA cells treated with solvent 0.1% dimethyl sulfoxide; Th—thapsigargin-treated HRA cells (ER-stressed HRA).

The mRNA expression of *ATF4*, *DDIT3* (which encodes for CHOP), *BAX* and *Bcl-2* was assessed in HRA cells treated with LDN-0060609 PERK inhibitor (0.75 μM and 25 μM), and HRA cells under ER stress also treated with LDN-0060609 (0.75 μM and 25 μM). The cells treated with both Th and 25 μM LDN-0060609 PERK inhibitor demonstrated a significant decrease in mRNA expression of the ER stress-related pro-apoptotic genes *ATF4, DDIT3* and *BAX* compared with those treated with Th alone. However, the ER-stressed HRA cells treated with 25 μM LDN-0060609 exhibited significantly greater mRNA expression of the anti-apoptotic *Bcl-2* gene compared to the untreated ER-stressed cells (Figure 2).

### 2.3. Assessment of the Potential Cytotoxic Acivity of the LDN-0060609 Compound 

The cytotoxicity of the tested small-molecule LDN-0060609 compound was measured using the 2,3-bis-(2-methoxy-4-nitro-5-sulfophenyl)-2H-tetrazolium-5-carboxanilide colorimetric assay (XTT assay). No significant cytotoxicity toward HRA cells was observed at any of the LDN-0060609 concentrations after 16, 24 or 48 h. Additionally, 0.1% DMSO, i.e., the used solvent, did not evoke significant cytotoxicity in HRA cells following the 16, 24 or 48 h incubation. 

**Figure 3:**
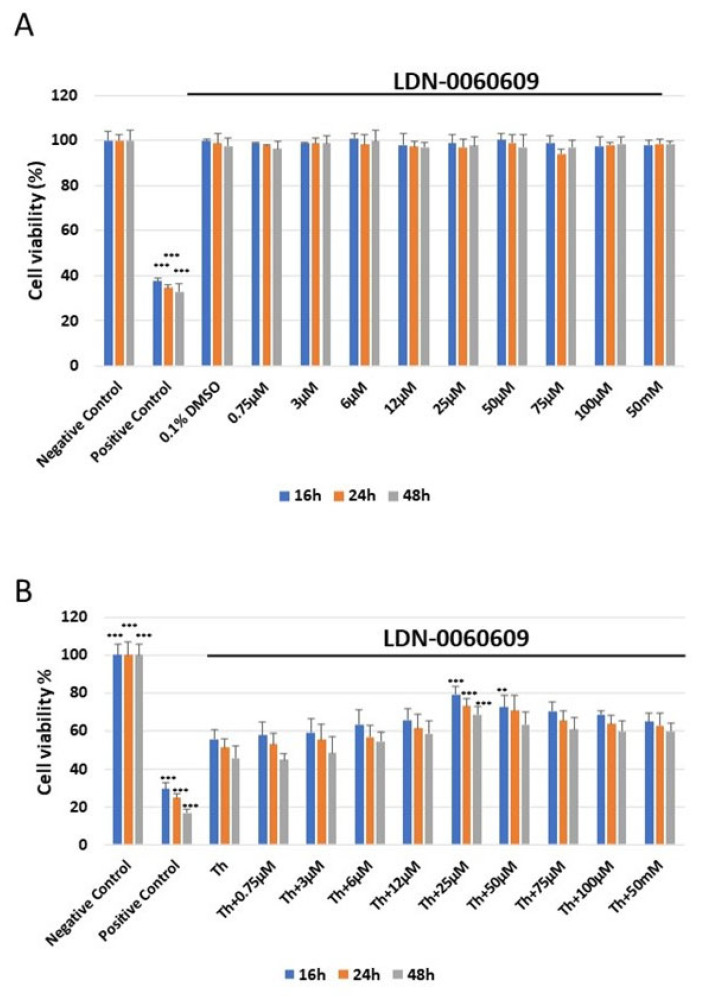
Analysis of the cytotoxicity of the LDN-0060609 compound toward HRA cells (**A**) and viability of ER-stressed HRA cells upon treatment with LDN-0060609 PERK inhibitor (**B**), as assessed by the colorimetric XTT assay. All experiments were performed in triplicate; values are expressed as mean ± SEM, *n* = 3. ** *p* < 0.01; *** *p* < 0.001 versus negative control (**A**) and Th (**B**). Negative control—untreated HRA cells; positive control—HRA cells treated with 99.9% dimethyl sulfoxide; 0.1% DMSO—HRA cells treated with the solvent, 0.1% dimethyl sulfoxide; Th—thapsigargin-treated HRA cells (ER-stressed HRA).

The cytotoxic effect of LDN-0060609 was also assessed in ER-stressed HRA cells. Treatment with Th for 16, 24 and 48 h showed a significant decrease in the percentage of viable HRA cells in comparison with the negative control. Interestingly, treatment of ER-stressed HRA cells with 25 μM LDN-0060609 significantly increased cell viability compared with untreated ER-stressed HRA cells at all incubation times (Figure 3).

### 2.4. Evaluation of the Genotoxic Poential of the LDN-0060609 Compound 

**Figure 4:**
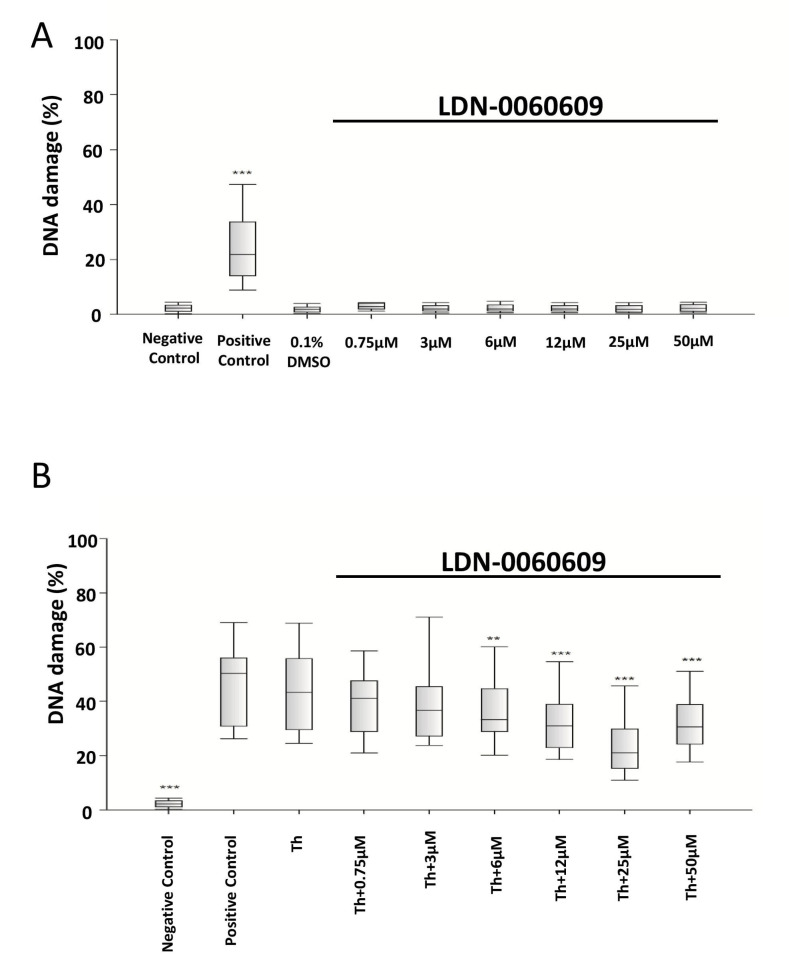
Genotoxicity analysis in HRA cells treated with the LDN-0060609 PERK inhibitor (**A**) and in ER-stressed HRA cells treated with LDN-0060609 (**B**), as indicated by the comet assay. All experiments were performed in triplicate. The value of cells scored for individual experiments was 100. Box plots show the median, first and third quartiles, minimum and maximum values. ** *p* < 0.01; *** *p* < 0.001 versus the negative control (**A**) and versus Th (**B**). Negative control—untreated HRA cells; Positive control—HRA cells treated with 99.9% dimethyl sulfoxide; 0.1% DMSO—HRA cells treated with the solvent, 0.1% dimethyl sulfoxide; Th—thapsigargin-treated HRA cells (ER-stressed HRA).

The level of DNA damage induced by LDN-0060609 was evaluated using an alkaline version of the comet assay. Neither LDN-0060609 nor its solvent, 0.1% DMSO, induced significant DNA damage in HRA cells at either concentration after 24 h incubation. A significant increase in DNA damage was demonstrated in ER =-stressed HRA cells compared with the negative control. Interestingly, after 24 h incubation, the ER-stressed HRA cells treated with 25 µM LDN-0060609 exhibited a greater reduction in DNA damage than the untreated ER-stressed HRA cells (Figure 4).

### 2.5. Analysis of the Level of Apoptosis in HRA Cells Using a Caspase-3 Assay

**Figure 5:**
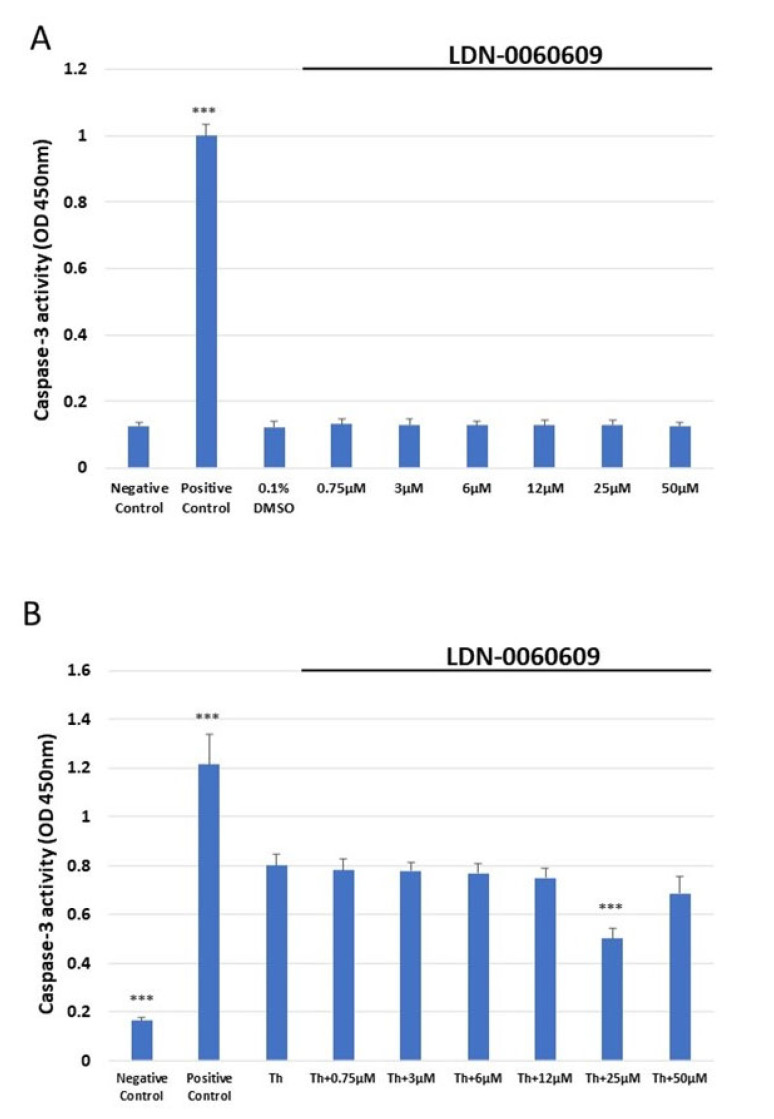
Assessment of the level of apoptosis in HRA cells (**A**) or ER-stressed HRA cells (**B**) following exposure to LDN-0060609. Results were determined by a caspase-3 assay. All experiments were performed in triplicate; values are expressed as mean ± SEM, *n* = 3. *** *p* < 0.001 versus the negative control (**A**) and versus Th (**B**). Negative control—untreated HRA cells; Positive control—HRA cells treated with 1 μM staurosporine; 0.1% DMSO—HRA cells treated with the solvent, 0.1% dimethyl sulfoxide; Th—thapsigargin-treated HRA cells (ER-stressed HRA).

Caspase-3 activity was determined in HRA cells treated with the LDN-0060609 PERK inhibitor using the colorimetric caspase-3 assay. It was found that 16 h exposure to 1 μM staurosporine significantly increased caspase-3 activity in HRA cells in comparison with negative controls, i.e., untreated HRA cells. In addition, no significant increase in caspase-3-dependent apoptosis was noted following 24 h exposure of HRA cells to LDN-0060609 (0.75–50 μM) or its solvent, 0.1% DMSO. Moreover, ER-stressed HRA cells showed significantly higher caspase-3 activity than the control HRA cells. The ER-stressed HRA cells demonstrated markedly decreased caspase-3 activity following treatment with 25 μM LDN-0060609 compared with the untreated ER-stressed cells (Figure 5).

### 2.6. Assessment of the Effect of LDN-0060609 on Cell Cycle Distribution and Progression in HRA Cells

**Figure 6:**
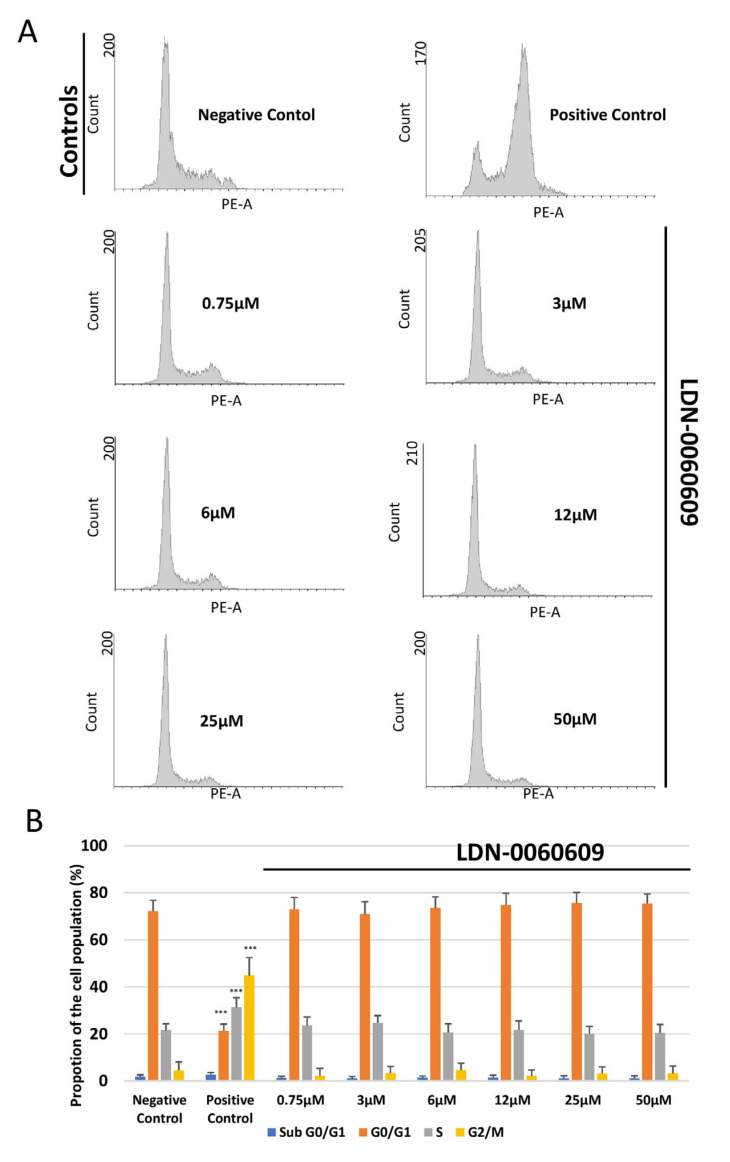

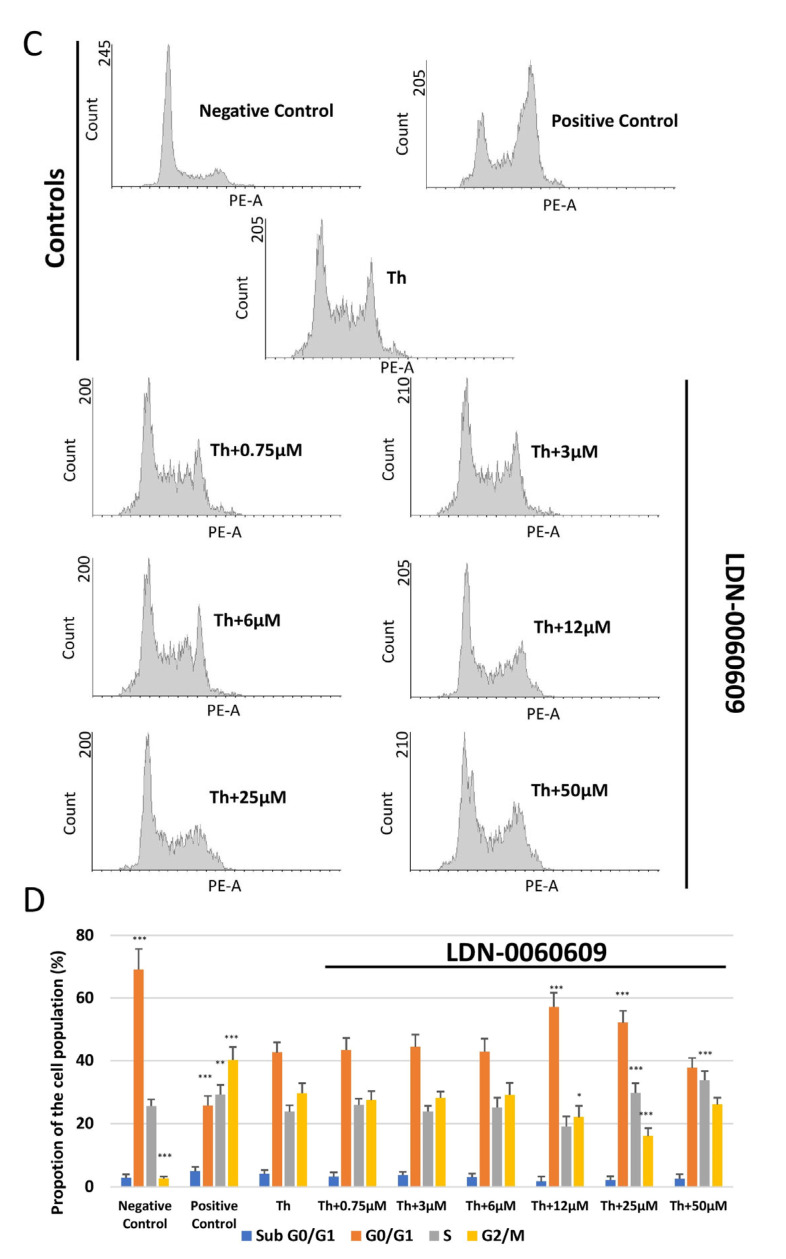
Flow cytometric analysis with propidium iodide (PI) staining of cell cycle progression in HRA cells exposed to LDN-0060609 alone (**A**,**B**) and ER-stressed HRA cells exposed to LDN-0060609 (**C**,**D**). The results are presented as histograms of DNA content (**A**,**C**) and bar graphs showing cell cycle distribution determined from the DNA content histograms (**B**,**D**). All experiments were performed in triplicate; values are expressed as mean ± SEM, *n* = 3. * *p* < 0.05, ** *p* < 0.01, *** *p* < 0.001 versus the negative control (**B**) and versus Th (**D**). Negative control—untreated HRA cells; Positive control—HRA cells treated with 1 μM nocodazole; Th—thapsigargin-treated HRA cells (ER-stressed HRA).

The cell cycle distribution and progression of HRA cells following treatment with LDN-0060609 was determined by flow cytometry with propidium iodide (PI) staining. It was found that neither LDN-0060609 alone (0.75–50 μM) nor the inhibitor solvent (0.1% DMSO) significantly affected HRA cell cycle distribution or progression, as compared to the negative control HRA cells. No significant differences were noted between the proportion of cells in the G0/G1, S and G2/M phases. The positive control (HRA cells exposed to 1 μM nocodazole) demonstrated a significant induction of cell cycle arrest in the G2/M phase as compared to the negative control (untreated HRA cells) (Figure 6A,B). Furthermore, in ER-stressed HRA cells, a higher proportion of cells were observed in the G2/M phase in comparison with the negative control. In addition, the ER-stressed HRA cells treated with 25 μM LDN-0060609 demonstrated a significantly lower percentage of cells in the G2/M phase compared to untreated ER-stressed HRA cells (Figure 6).

## 3. Discussion

Astrocytes are a major component of the ONH, where they provide structural and biochemical support for the unmyelinated axons of RGCs. Recently, interest in astrocyte function has grown, as their activation has been shown to precede axonal damage [24,25], suggesting that their dysfunction may be a central aspect of glaucoma development. However, the role of astrocyte reactivity in maintaining RGC survival and visual function remains ambiguous. Several reports indicate that reactive astrocytes constitute a major driver of RGC death, and suppression of their reactive phenotype may alleviate RGC survival [26,27]; however, a substantial body of evidence suggests that activated astrocytes alleviate neurodegenerative stress, contributing to the survival of RGC and the maintenance of visual function, while impairment of astrocyte function represents a significant contributor to glaucoma progression [28,29,30]. Additionally, recent studies based on induced pluripotent astrocytes from a glaucoma patient suggest that astrocytes influence glaucoma progression more by taking a diminished neuro-supportive role than adopting a neurotoxic phenotype [31].

Recent studies indicate that excessive activation of the PERK-dependent UPR pathway generates a distinct state of reactivity in CNS astrocytes, characterized by the depletion of the astrocytic secretome and a loss of neuroprotection [32]. Furthermore, it has been proposed that increased PERK-dependent signaling occurring in response to elevated IOP or a hypoxic environment in the ONH may lead to unfavorable activation of retinal astrocytes [33,34]. A study by Kazuki Ojino et al. conducted on a DBA/2J mouse model of glaucoma found that the ER stress-related proteins BiP and CHOP were predominantly found in reactive astrocytes adjacent to damaged nerve fibers, suggesting that ER stress-related events might exacerbate the deleterious effect of reactive astrocytes on RGC axons [33]. Another study found that reactive astrocytes exhibit increased CHOP expression in response to a hypoxic environment, and colocalize within the unmyelinated portion of the optic nerve, the site most vulnerable to glaucomatous damage. Importantly, UPR-mediated activation of astrocytes was found to occur before the loss of RGCs [34], indicating that astrocytes may represent the first-line target for UPR inhibition, even before the onset of axonal degeneration. Finally, persistent CHOP expression has been shown to cause astrocyte death, which may be indicative of an irreversible loss of metabolic support for RGC axons, resulting in optic nerve degeneration [35,36]. To date, the vast majority of studies on the role of astrocytes in the field of neurodegeneration have originated from mouse models, and recent data indicate a significant divergence in sensitivity and response to stressors between human and mouse astrocytes [37]. Therefore, the present study has focused on astrocytes derived from the human retina.

Our findings indicate that treatment of HRA cells with Th significantly elevated the phosphorylation of eIF2α, the main PERK substrate, implying activation of ER stress conditions and PERK-mediated signaling. The investigated PERK inhibitor was able to markedly inhibit eIF2α phosphorylation in HRA cells upon ER stress induction and significantly decrease the expression of pro-apoptotic genes such as *ATF4*, *DDIT3*, and *BAX*. Moreover, the investigated compound did not induce any genotoxic or cytotoxic effect in HRA cells at any used concentration, and at 25 μM, it reduced DNA damage and increased the viability of HRA cells treated with Th. Furthermore, our data demonstrate that the investigated PERK inhibitor did not affect cell apoptosis when used alone, nor did it influence the cell cycle progression or distribution in HRA cells at any given concentration. Importantly, at 25 μM, LDN-0060609 prevented cell apoptosis and decreased the percentage of HRA cells in G2/M after ER stress induction, compared to those treated with Th alone. Recent reports highlight the importance of preserving astrocyte viability and maintaining protein homeostasis as a promising strategy for neuroprotection in glaucoma [38]. Our present findings demonstrate that the use of LDN-0060609 protects HRA cells from apoptosis and restores protein homeostasis; they therefore also suggest that the compound may alleviate the negative effects of ER stress in HRA, and thus provide a novel treatment strategy for POAG.

A comprehensive assessment of the role of astrocytes in the pathogenesis of POAG is complicated by the heterogeneity of astrocyte populations in the retina, optic nerves, and brain [39]. In addition, mouse retinal astrocytes demonstrate varied region-specific and temporally distinct transcriptional activities in response to elevated intraocular pressure [38]. Furthermore, reactive astrocytes can be polarized into pro-inflammatory neurotoxic A1 or anti-inflammatory neuroprotective A2 states, further complicating their responses to local stressors. Indeed, rather than two different populations, astrocytes may exist as a continuum of A1 and A2 phenotypes moving within a dynamic range of functional characteristics [40]. As a result, it may be difficult to distinguish the impacts of one functional phenotype of astrocytes involved in the development of glaucoma from another. More research is needed to identify suitable astrocyte-based biomarkers and their effects on pathological hallmarks in glaucoma models. Future studies could also be conducted to investigate how PERK inhibition affects the phenotype of astrocytes. 

The effectiveness of the investigated small-molecule PERK inhibitor has been successfully confirmed in primary human trabecular meshwork (HTM) cells with thapsigargin-induced ER stress as a cellular model of POAG [41]; these findings have raised the prospect of extending its research to retinal astrocytes. However, the present work is the first such study to evaluate the outcomes of PERK-dependent pathway inhibition in the context of human retinal astrocytes.

## 4. Materials and Methods

### 4.1. The Identification of the LDN-0060609 PERK Inhibitor

The small-molecule PERK inhibitor LDN-0060609 was screened and characterized according to Pytel et al. (2014) [42]. Briefly, the inhibitor was selected from the Laboratory for Drug Discovery in Neurodegeneration (LDDN) compound library via high-throughput assay. The compound was then provided for further analysis by the Department of Biochemistry and Molecular Biology, Hollings Cancer Center, Medical University of South Carolina, Charleston, SC, USA.

### 4.2. Cell Culture

The experiments were conducted in vitro using a commercially available human retinal astrocyte (HRA) cell line isolated from a healthy human retina. The HRA cells were obtained from Innoprot (Innoprot [Innovative Technologies in Biological Systems], Derio, Spain). The cell culture was maintained in accordance with the manufacturer’s protocol, under standard cell culture conditions (37 °C; 5% CO_2_; 95% humidity), and cultured in recommended Astrocyte Medium (AM) containing astrocyte basal medium (BM), 2% fetal bovine serum (FBS) (Innoprot), 1% astrocyte growth supplement (Innoprot) and 1% penicillin/streptomycin solution (P/S) (Innoprot). The cells were cultured on T-75 culture vessels coated with poly-L-lysine (2 μg/cm^2^). HRA cells were split every 3–4 days, upon reaching the 90–95% confluency, and after exposure to 0.25% trypsin/EDTA (T/E) solution (Innoprot).

### 4.3. Western Blotting

The ER-stressed HRA cells were exposed to LDN-0060609 and tested for PERK enzyme activity by measuring eIF2α phosphorylation, the major substrate of PERK. The protein expression of p-eIF2 α, eIF2α and β-actin was detected by Western blotting. Each experiment was repeated three times with similar results. 

Briefly, HRA cells were pre-incubated for one hour with LDN-0060609 at a range of concentrations (0.75–50 μM) and then exposed to an ER stress inducer, Th, at 500 nM and incubated for two hours. Cells incubated with 500 nM Th alone for two hours (i.e., those under ER stress) were used as positive controls, and the cells cultured in complete AM cell culture medium alone were used as negative controls. 

Following this, the total protein was extracted by means of a MinuteTM Total Protein Extraction Kit (Invent Biotechnologies Inc., Plymouth, MI, USA) using native cell lysis buffer (SN-002) supplemented with the Protease and Phosphatase Inhibitor Cocktail (Thermo Fisher Scientific Inc., Waltham, MA, USA). A standard Bradford assay was performed to measure the protein concentration, with bovine serum albumin (BSA) used as a protein standard. Following this, 85µg of protein from each sample was mixed with 2 X SDS-PAGE loading buffer (Bio-Rad Laboratories, Hong Kong, China) and boiled for 10 min at 95 °C. 

The samples were resolved by 10% sodium dodecyl sulfate-polyacrylamide gel electrophoresis (SDS-PAGE), transferred onto a microporous polyvinylidene difluoride (PVDF) membrane (Immobilon-P, Merck Millipore, Burlington, MA, USA), blocked for one hour at room temperature (RT) in 5% *w*/*v* non-fat milk in 1 X Tris-buffered saline and 0.1% Tween 20 (TBST), and incubated overnight at 4 °C with primary antibodies against p-eIF2α and eIF2α (Cell Signaling Technology Inc., Danvers, MA, USA) diluted in 5% *w*/*v* non-fat milk, 1 X TBST. The β-actin antibody (Cell Signaling Technology Inc.) served as a loading control. After probing with HRP-conjugated secondary antibody (Cell Signaling Technology Inc.) diluted in 5% *w*/*v* non-fat milk, 1 X TBST at RT for one hour, the protein bands were visualized using an enhanced chemiluminescence detection kit (Bio-Rad Laboratories). Following the visualization, the X-ray films were scanned and protein band intensity was quantified using Gene Tools 4.3.17 software (Syngene, Cambridge, UK).

### 4.4. Assesment of the Expression of the ER Stress-Related Genes

Total RNA was isolated from the HRA cells using a PureLink RNA Mini Kit (Thermo Fisher Scientific Inc.) in accordance with the manufacturer’s instructions. The isolated RNA was then transcribed into cDNA by GoScript ™ Reverse Transcriptase (Promega Inc., Madison, WI, USA) in accordance with the manufacturer’s protocol, to a final concentration of 100 ng. The expression profile of the following ER stress-related apoptotic genes was determined using TaqMan Gene Expression Assays: *ATF4* (Hs00909569_g1), *BAX* (Hs00180269_m1), *Bcl-2* (Hs00608023_m1) and *DDIT3* (Hs01090850_m1). *ACTB* (Hs99999903_m1) was used as a reference gene. The total reaction volume for the qPCR analysis was 10 µL, and it included the following reagents: 1 µL primers, 1 uL cDNA, 2 µL 5x HOT FIREPol^®^ Probe qPCR Mix (Solis BioDyne, Tartu, Estonia), and 6 µL nuclease-free water. The reaction conditions were set as described in the manufacturer’s protocol: enzyme activation (15 min, 95 °C), denaturation (40 cycles, 10 s, 95 °C), and annealing/extension (40 cycles, 60 s, 60 °C). The gene expression level was assessed with the Bio-Rad CFX96 (BioRad Laboratories) system.

### 4.5. Cytotoxicity Analysis

The cytotoxicity of the analyzed LDN-0060609 compound was evaluated using the colorimetric XTT assay (Thermo Scientific), which detects cell metabolic activity. During the test, the metabolically active cells reduce the yellow tetrazolium salt XTT to an orange formazan dye via dehydrogenase enzymes. All experiments were performed three times with similar results. HRA cells were seeded in a 96-well plate (5 × 10^3^/well) coated with poly-L-lysine and cultured for 24 h in 100 μL of complete AM growth medium. Following this, the cells were exposed to 100 μL of complete medium containing LDN-0060609 at a wide range of concentrations (0.75 μM, 3 μM, 6 μM, 12 μM, 25 μM, 50 μM, 75 μM, 100 μM, 50 mM) or the solvent for LDN-0060609, 0.1% DMSO (Sigma-Aldrich Corp., St. Louis, MO, USA). The negative control consisted of HRA cells cultured only in complete medium, and the positive controls were cells incubated with 100% DMSO. 

Additionally, to assess the effect of the LDN-0060609 inhibitor in ER-stressed HRA cells, the HRA cells were seeded in 96-well plates (5 × 10^3^/well) coated with poly-L-lysine and cultured for 24 h in 100 μL of complete AM growth medium. Following this, the cells were preincubated with 100 μL of complete medium containing LDN-0060609 at a range of concentrations (0.75-100 μM and 50 mM) for one hour. The HRA cells were then exposed to Th (500 nM) to evoke ER stress conditions. Some cells were treated only with Th at 500 nM. The negative controls consisted of untreated HRA cells cultured in a complete medium, and the positive controls of cells incubated with 100% DMSO. All the analyzed samples were incubated with the respective compounds or media for 16, 24 or 48 h. Next, 25 µL of XTT/PMS mixture was added to each well, following the manufacturer’s instructions. After two-hour incubation in a 5% CO_2_ incubator at 37 °C, the absorbance was recorded at a wavelength of 450 nm using Synergy HT spectrophotometer (BioTek, Winooski, VT, USA).

### 4.6. Genotoxicity Analysis

The genotoxicity of LDN-0060609 PERK inhibitor was determined using an alkaline comet assay (ACA), which is a sensitive method for detecting DNA strand breakage. All experiments were performed three times with similar results. For the assay, the cells were seeded at 2 × 10^5^ in poly-L-lysine-coated 6-well plates in 2 mL of complete AM cell culture medium. Next, the cells were exposed to LDN-0060609 at a wide range of concentrations (0.75–50 μM) or the solvent, 0.1% DMSO (Sigma-Aldrich Corp.) and incubated for 24 h. Positive controls consisted of cells suspended in 5% DMSO (Sigma-Aldrich Corp.), and negative controls of cells cultured only in complete AM cell culture medium. 

In addition, the effect of LDN-0060609 was analyzed in ER-stressed HRA cells. In this experiment, HRA cells were seeded in 6-well plates coated with poly-L-lysine at 2 × 10^5^ in 2 mL of complete AM cell culture medium and cultured for 24 h. Subsequently, the HRA cells were pre-incubated for one hour with 2 mL of complete medium containing LDN-0060609 at concentrations ranging from 0.75 to 50 μM. Then, HRA cells were treated with 50 nM Th. Some cells were treated only with Th at 50 nM. The positive controls consisted of HRA cells suspended in 5% DMSO (Sigma-Aldrich Corp.), and the negative controls of cells suspended in 2 mL of complete medium. Following the treatment, all the samples were incubated for 24 h. 

The cells were suspended in 0.37% low-melting-point (LMP) agarose (Sigma-Aldrich Corp.), which was placed on microscope slides pre-coated with normal-melting-point (NMP) agarose (Sigma-Aldrich Corp.). The preparations were incubated in pH 10 lysis buffer (2.5 M NaCl, 10 mM Tris, and 100 mM EDTA) and TritonX-100 (Sigma-Aldrich Corp.) at a final concentration of 1% for one hour at 4 °C. 

After the lysis, the preparations were incubated in development buffer (300 mM NaOH and 1 mM EDTA) for 20 min at 4 °C, and then the samples were subjected to electrophoresis (32 mA, 17 V, 20 min) in the electrophoretic buffer (30 mM NaOH and 1 mM EDTA) at 4 °C. Next, the preparations were rinsed with distilled water three times and left to dry at RT. Then, the preparations were stained with a fluorescent dye (DAPI) and analyzed under a fluorescence microscope. The DNA damage in cells was measured by the percentage of DNA content in the comet tail.

### 4.7. Apoptosis Analysis

The activation of apoptosis in HRA cells was analyzed using a caspase-3 assay kit (Abcam), i.e., a colorimetric assay detecting the activity of caspase-3 in cell lysates. All conducted experiments were repeated three times with similar results. HRA cells were seeded in 6-well plates coated with poly-L-lysine (5 × 10^5^/well) and cultured in a complete AM cell culture medium for 24 h. Subsequently, the cells were treated with LDN-0060609 at a concentration of 0.75 to 50 μM or with the solvent 0.1% DMSO (Sigma-Aldrich Corp., St. Louis, MO, USA) for 24 h. The positive controls consisted of cells treated with 1 µM of staurosporine (Sigma-Aldrich Corp., St. Louis, MO, USA) for 16 h, and the negative controls of cells incubated in complete AM medium for 24 h. 

To investigate the effect of LDN-0060609 on ER-stressed HRA cells, the cells were seeded in 6-well plates coated with poly-L-lysine (5 × 10^5^/well) and cultured for 24 h in complete medium. Next, the cells were preincubated for one hour with the complete medium containing LDN-0060609 at concentrations from 0.75 to 50 μM, before being exposed to 500 nM Th for 24 h. Some HRA cells were treated only with Th (500 nM, 24 h). The positive controls consisted of cells treated with 1 µM staurosporine (Sigma-Aldrich Corp., St. Louis, MO, USA) for 16 h, and the negative controls of cells cultured for 24 h alone in complete AM medium.

Next, the cells were washed once with 1 X DPBS (Sigma-Aldrich Corp., St. Louis, MO, USA) and detached with 0.25% T/E solution (Innoprot). Subsequently, the obtained cell suspension was centrifuged for 5 min at 1000 rpm at RT, and the supernatant was removed. The cells were then resuspended in a complete AM medium, counted and centrifuged at for 5 min 1000 rpm at RT. The obtained pellet containing 1 × 10^6^ cells was resuspended in 50 µL of cold Cell Lysis Buffer and incubated on ice for 10 min. The cell suspension was then centrifuged for 1 min at 10,000× *g*. The obtained supernatants were then transferred to fresh 2 mL tubes. 

The protein concentration was measured by a standard Bradford assay, with BSA used as a protein standard. For each assay, the cell lysate containing 100 μg protein was used. 2 X Reaction Buffer containing 10 mM DTT and 4 mM DEVD-pNA substrate (200 µM final concentration) was then added to each sample, and the samples were incubated for two hours at 37 °C. After the incubation, the p-NA absorbance was detected at 405 nm wavelength using a Synergy HT spectrophotometer (BioTek).

### 4.8. Cell Cycle Analysis

The cell cycle analysis was carried out using PI staining with flow cytometry. All experiments were repeated three times with similar results. The HRA cells were seeded in 6-well plates coated with poly-L-lysine (5 × 10^5^/well) and cultured for 24 h in a complete AM cell culture medium. Then, HRA cells were incubated for 24 h with the LDN-0060609 at concentrations ranging from 0.75 to 50 μM or with 0.1% DMSO (Sigma-Aldrich Corp., St. Louis, MO, USA). The positive controls consisted of HRA cells exposed to 1 µM nocodazole (Sigma-Aldrich Corp., St. Louis, MO, USA) and incubated for 16 h, and the negative controls of cells cultured for 24 h in a complete AM medium. 

In order to assess the effect of LDN-0060609 in ER-stressed HRA cells, the cells were seeded in 6-well plates coated with poly-L-lysine (5 × 10^5^/well) and cultured for 24 h in a complete AM cell culture medium. After that, the cells were pretreated for one hour with the complete medium containing LDN-0060609 at concentrations ranging from 0.75 to 50 μM, and then the HRA cells were exposed to 500 nM Th for 24 h. Some cells were treated with Th alone (500 nM, 24 h). The positive controls consisted of cells incubated with nocodazole at 1 µM (Sigma-Aldrich Corp., St. Louis, MO, USA) for 16 h, and the negative controls of cells cultured for 24 h in complete medium. 

Subsequently, the HRA cells were harvested and double-washed with cold 1 X DPBS (Sigma-Aldrich Corp.). The cells were counted and the amount of 1 × 10^6^ cells/mL was fixed with ice-cold 70% ethanol for 20 min at −20 °C. The ethanol-suspended cells were then centrifuged for 5 min at 5000 rpm. The cell pellets were suspended in 250 µL of 1 X DPBS (Sigma-Aldrich Corp., St. Louis, MO, USA), treated with RNase A, DNase and protease-free water at 10 mg/mL (Canvax Biotech, Valladolid, Spain), incubated for one hour at 37 °C, and then stained with 10 μg/mL PI solution (Sigma-Aldrich Corp., St. Louis, MO, USA). After a further 30 min incubation at 4 °C, the samples were finally analyzed using a Beckman Coulter CytoFLEX. The percentage of cells at each phase of the cell cycle was determined by Kaluza analysis software version 1.5 A (Beckman Coulter, Brea, CA, USA), based on the DNA content measured by PI fluorescence. In the histograms of DNA content, the number of cells was depicted on the y-axis, and the DNA content was plotted on the x-axis.

### 4.9. Statistical Analysis

The statistical analysis was carried out using Sigma Plot software (version 11.0; Systat Software Inc., San Jose, CA, USA). In all conducted experiments, all continuous variables were tested for normality using the Shapiro–Wilk test. As the data in all experiments were normally distributed (except the comet assay), the Student’s *t*-test was used to compare pars of groups. In the comet assay, where no normal distribution was obtained, the pairs of groups were compared using the Mann–Whitney U-test. The statistical analysis in each individual experiment was based on the results of three independent tests. In the graphs, statistically significant differences were indicated as follows: * *p* < 0.05, ** *p* < 0.01, *** *p* < 0.001.

## 5. Conclusions

Glaucoma is estimated to affect more than 111 million people worldwide by the end of 2040. The disease is often called the “silent thief of sight”, as it slowly and painlessly leads to permanent loss of vision. Recent observations suggest that HMA cells play a key role in glaucoma pathology, as it has been found that chronic ER stress induces apoptosis of HRA cells via induction of the PERK/DDIT3 axis of the UPR; this abolishes their neuroprotective potential and leads to glaucoma progression. The present study evaluates the potential of the small-molecule PERK inhibitor LDN-0060609 to counter ER stress conditions induced in HRA cells in vitro. Western blot analysis revealed that LDN-0060609 at 25 μM effectively inhibited the PERK-dependent phosphorylation of eIF2α (~50%). Further, the tested compound at the same concentration markedly decreased the expression levels of pro-apoptotic ER stress-related genes, namely *ATF4, DDIT3, BAX* and *Bcl-2*, in ER-stressed HRA cells. Treatment of ER-stressed HRA cells with 25 μM LDN-0060609 significantly increased cell viability, decreased DNA damage and caspase-3 level, and restored cell cycle distribution. At the same time, the compound was not cytotoxic or genotoxic towards HRA cells at any concentration or incubation time, nor did it affect protein expression or mRNA expression of caspase-3 level in HRA cells; it also did not influence cell cycle progression or distribution. Altogether, or findings indicate that the small-molecule PERK inhibitor LDN-0060609 plays a protective role in an in vitro model of glaucoma by alleviating negative effects related to ER stress in HRA cells. It may thus serve as a new strategy for treating POAG.

## 6. Patents

Patent no. Pat.241218, granted by The Polish Patent Office.

## Data Availability

The data that support the findings of this study are available from the corresponding author upon reasonable request.
